# Pressure dependence of metal–silicate partitioning explains the mantle phosphorus abundance

**DOI:** 10.1038/s41598-024-51662-y

**Published:** 2024-01-12

**Authors:** Nagi Ikuta, Naoya Sakamoto, Shoh Tagawa, Kei Hirose, Yutaro Tsutsumi, Shunpei Yokoo, Hisayoshi Yurimoto

**Affiliations:** 1 Department of Earth and Planetary Science, The University of Tokyo, Tokyo, 113-0033 Japan; 2 Creative Research Institution (CRIS), Hokkaido University, Sapporo, Hokkaido 001-0021 Japan; 3 Earth-Life Science Institute, Tokyo Institute of Technology, Tokyo, 150-8550 Japan; 4 Department of Natural History Sciences, Hokkaido University, Sapporo, Hokkaido 060-0810 Japan

**Keywords:** Geochemistry, Geochemistry

## Abstract

Previous experiments performed below 20 GPa suggested that the metal/silicate partition coefficient of phosphorus (P), *D*_P_, extrapolated to typical high-pressure and -temperature conditions of the Earth’s core formation gives too high P concentration in the core unless a large amount of silicon was included in metals. Here we examined *D*_P_ between liquid metal and coexisting molten silicate at 27–61 GPa and 3820–4760 K, corresponding to conditions of core-forming metal segregation from silicate, by measuring recovered samples using a high-resolution imaging technique coupled with secondary ion mass spectrometry. The results demonstrate that the pressure dependence of *D*_P_ changes from positive to negative above 15 GPa, likely because of an increase in the coordination number of P^5+^ in silicate melt. With the present new partitioning data, the observed mantle P abundance may indicate ~ 0.2 wt% P in the core, consistent with the cosmo-/geochemical estimates, based on both single-stage and multi-stage core formation models without involving high amounts of silicon in metals.

Phosphorus is moderately siderophile and could be one of important light elements in planetary cores^1^. The metal/silicate partition coefficients of phosphorus *D*_P_ have been extensively studied below 3 GPa and previously reported up to 20 GPa and 2873 K using multi-anvil apparatuses^2–12^ (Supplementary Table S1). These earlier experiments demonstrated that the relatively high P abundance in the Martian mantle is explained by metal–silicate partitioning at the base of a magma ocean under relatively low pressure–temperature (*P*–*T*) and high oxygen fugacity (*f*O_2_) conditions (6–10 GPa, 1900–2200 K and ΔIW–1.5 to − 1)^11^. On the other hand, extrapolation of these low *P*–*T* data to typical high *P*–*T* conditions of the Earth’s core formation (30–60 GPa, 2500–4000 K, ΔIW = − 2.3) shows *D*_P_ > 1300 (mole based)^6^. With such high *D*_P_ value, P concentration in the mantle (80–90 ppm)^13,14^ suggests > 11 wt% P in the core, which almost explains the core density deficit without any additional light elements^1^ and is thus unlikely. Alternatively Righter et al.^9^ demonstrated that when metals are enriched in silicon (> 10 wt%), *D*_P_ is sufficiently low and gives little P abundance in the core. The > 10 wt% Si is, however, more than required to explain the core density deficit and velocity excess^15^.

Indeed, extrapolation of existing metal–silicate partitioning data to higher pressures may not be straightforward. Cottrell et al*.*^16^ demonstrated that the pressure evolution of the metal–silicate partitioning of tungsten, a high-field strength element similar to phosphorus, changes from positive to negative above ~ 3 GPa. Rai and van Westrenen^17^ also showed that the pressure dependence of the metal–silicate partitioning of Ni, Co, Mo, V and Cr also changes above 5 GPa. A clear change has not been found for P, but it could be because high-pressure data (> 5 GPa) were limited (Supplementary Table S1).

In this study, we explore the metal–silicate partitioning of phosphorus to *P*–*T* conditions much higher than in earlier experiments (Table 1). Low P concentrations in quenched silicate melts were determined with high-resolution SIMS analyses. Combining with previous low *P*–*T* experimental data, we found that the pressure dependence changes from positive to negative above 15 GPa. 80–90 ppm P observed in the Earth’s mantle may suggest ~ 0.2 wt% P in the core when considering metal–silicate partitioning in a deep magma ocean in both single-stage and multi-stage core formation scenarios, consistent with cosmo-/geochemical estimates of the bulk Earth P abundance^18,19^.

**Table 1:** Summary of the present experiments.

Run #	1	2	3	4	5	6
*P* (GPa)	45 (5)	56 (6)	60 (6)	34 (3)	61 (6)	27 (3)
*T* (K)	4410 (220)	4260 (210)	4760 (240)	3820 (190)	4560 (230)	4060 (200)
P in metal by EPMA (wt %)	2.25 (28)	3.75 (7)	1.96 (10)	2.61 (26)	1.96 (10)	2.16 (40)
P in silicate by SIMS (ppm wt)	133 (45)	39 (17)	87 (36)	42 (27)	206 (106)	266 (38)
*D*_P_ (weight based)	169 (61)	960 (407)	226 (93)	615 (390)	95 (49)	81 (19)
*D*_P_ (mole based)	164 (60)	918 (389)	234 (97)	564 (358)	94 (49)	73 (19)
*f*O_2_ (ΔIW)	− 1.29	− 1.92	− 1.69	− 1.37	− 1.62	− 0.99
*nbo*/*t*	1.15	0.65	0.75	1.18	0.69	1.49

Numbers in parentheses indicate one standard deviation in the last digits.

## Results

**Figure 1:**
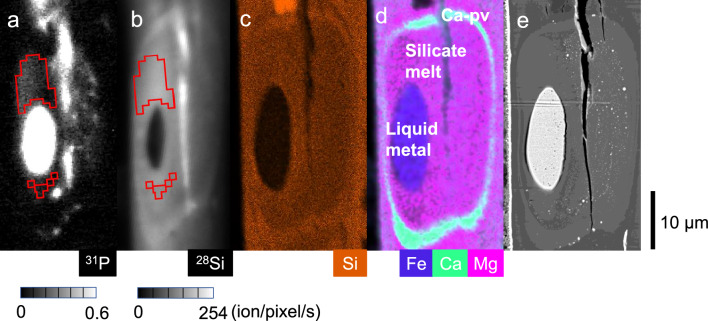
Secondary ion images for (**a**) ^31^P^−^ and (**b**) ^28^Si^−^, EDS X-ray maps for (**c**) Si and (**d**) Fe/Ca/Mg, and (**e**) back-scattered electron image of a sample cross section obtained in run #3. Quenched liquid metal was surrounded by silicate melt. Ca-rich silicate (labelled as Ca-pv) with a composition similar to that of CaSiO_3_ perovskite was present outside the silicate melt. Note that the images in (**c–e**) were obtained after imaging by SIMS (**a**, **b**) and repolishing by FIB technique. As a result, the area of liquid metal was enlarged. The liquid metal area of (**a**) is apparently larger than that of (**b**) because of lens-flare effects of the secondary ion optics due to extremely high ^31^P intensities from liquid metal (> 0.6 ion/pixel/s). Portions surrounded by red lines (ROI) in the silicate melt shown in (**a**, **b**) is free from the lens-flare effects (Supplementary Fig. S1). The reason why the secondary ion intensities of P and Si are apparently high along the crack is unknown, but probably due to some artifact effects of secondary ion emissions. Small liquid metal particles are scattered, especially in the right-hand side portion, in the silicate melt as shown in (**e**). Indeed, secondary ion intensities of P from the particles are high (**a**). From these petrographic analyses, the ROIs shown in (**a**, **b**) are assumed to be the silicate melt equilibrated with the liquid metal, to calculate the P content. P concentration in the ROI varies from 24 to 170 ppm, and the concentration in the silicate melt is determined to be 87 ± 36 ppm.

Six separate metal–silicate partitioning experiments were conducted at 27–61 GPa and 3820–4760 K in a diamond-anvil cell (DAC) (Table 1) (see “Methods” section). The chemical compositions of coexisting molten silicate and liquid metal are given in Table 2. We found 19,600 to 37,500 ppm P in liquid metal and only 39 to 266 ppm P in surrounding silicate melt (both by weight) (Fig. 1 and Supplementary Figs. S1 to S6), providing ${D}_{{\text{P}}}^{metal/silicate}$ (mole based) = 73 to 918 (see Supplementary Fig. S7 for relations between mole-based and weight-based *D*_P_). The metal–silicate partitioning of phosphorus can be expressed as a chemical reaction;

**Table 2:** Chemical compositions (wt%) of the silicate starting material, silicate melts and quenched liquid metals formed by melting experiments.

Run #		SM1	SM2	1	2	3	4	5	6
SM		–	–	1	1	1	1	1	2
Silicate	Num.^a^	6	6	6	6	6	6	6	8
	SiO_2_	51.19 (44)	48.59 (57)	41.32 (56)	40.24 (104)	44.10 (99)	43.51 (80)	43.23 (86)	37.98 (118)
	Al_2_O_3_	16.49 (24)	15.20 (11)	14.64 (62)	24.82 (55)	21.75 (26)	12.75 (109)	22.84 (86)	10.47 (56)
	FeO	9.46 (8)	9.40 (35)	25.78 (114)	11.99 (116)	16.45 (65)	22.72 (233)	17.05 (85)	28.36 (251)
	MgO	8.59 (6)	8.57 (7)	8.74 (29)	11.43 (17)	9.87 (19)	9.20 (66)	9.02 (35)	8.38 (48)
	CaO	4.94 (23)	9.36 (14)	3.45 (15)	4.41 (7)	4.83 (12)	4.71 (41)	4.02 (21)	5.29 (23)
	Na_2_O	4.20 (10)	3.40 (11)	3.96 (14)	4.98 (116)	5.37 (30)	4.18 (27)	5.53 (35)	2.40 (14)
	K_2_O	0.10 (1)	0.14 (2)	0.24 (2)	0.69 (5)	0.31 (1)	0.23 (1)	0.29 (6)	0.13 (3)
	TiO_2_	–	1.00 (6)	–	–	–	–	–	1.28 (9)
	Total	94.96 (64)	95.66 (65)	98.13 (55)	98.58 (82)	102.69 (61)	97.30 (83)	101.98 (48)	96.61 (62)
Metal	Num.^a^	–	–	6	6	6	12	6	20
	Fe	–	–	91.75 (90)	88.75 (29)	86.49 (34)	92.79 (117)	86.70 (206)	76.75 (238)
	Si	–	–	0.09 (4)	3.14 (16)	3.72 (21)	0.96 (31)	2.55 (24)	3.36 (101)
	O	–	–	1.85 (67)	2.40 (16)	2.15 (23)	2.28 (53)	3.67 (33)	5.18 (134)
	Al	–	–	ND	0.03 (2)	0.03 (0)	0.01 (1)	0.06 (1)	–
	Mg	–	–	ND	0.02 (1)	0.01 (1)	ND	0.02 (1)	–
	Ti	–	–	–	–	–	–	–	0.12 (5)
	C	–	–	1.58 (24)	1.42 (15)	1.61 (11)	0.82 (7)	1.15 (12)	2.31 (26)
	P	–	–	2.25 (28)	3.75 (7)	1.96 (10)	2.61 (26)	1.96 (10)	2.16 (41)
	Total	–	–	97.54 (61)	99.52 (17)	95.98 (34)	99.48 (65)	96.10 (153)	89.88 (77)

Numbers in parenthesis indicate one standard deviation in the last digits. ^a^Number of analyses.

1 $${{\text{PO}}}_{2.5}^{silicate\ melt}+\frac{5}{2}{{\text{Fe}}}^{metal}={{\text{P}}}^{metal}+\frac{5}{2}{{\text{FeO}}}^{silicate\ melt}$$

The exchange coefficient *K*_D_ for this reaction is parameterized as a function of *P*, *T* and *nbo*/*t* (Ref.^6^) with regression constants *a*, *b*, *c* and *d*;

2 $$\begin{aligned} \log_{10} K_{{\text{D}}} & = \log_{10} \frac{{{x^{\prime}}_{{\text{P}}}^{metal} }}{{x_{{\text{PO}_{2.5} }}^{silicate} }} \cdot \left( {\frac{{x_{{{\text{FeO}}}}^{silicate} }}{{{x^{\prime}}_{\text{Fe}}^{metal} }}} \right)^{\frac{5}{2}} = \log_{10} \frac{{{x^{\prime}}_{{\text{P}}}^{metal} }}{{x_{{\text{PO}_{2.5} }}^{silicate} }} + \frac{5}{4}\Delta {\text{IW}} \\ & = \log_{10} D_{{\text{P}}}^{metal/silicate} + \frac{5}{4}\Delta {\text{IW}} = a + \frac{b}{T} + c \cdot \frac{P}{T} + d \cdot \frac{nbo}{t} \\ \end{aligned}$$

where *x* and *x*′ represent molar fractions in silicate and metal, respectively (see “Supplementary text”), and *nbo*/*t* is an empirical parameter, the molar ratio of non-bridging oxygens per cations that are tetrahedrally coordinated at low pressures. Oxygen fugacity relative to the iron-wüstite (IW) buffer is approximated as $\mathrm{\Delta IW}\approx 2 \ {{\text{log}}}_{10}\left(\frac{{x}_{{\text{FeO}}}^{silicate}}{{{x^{\prime}}}_{{\text{Fe}}}^{metal}}\right)$.

**Figure 2:**
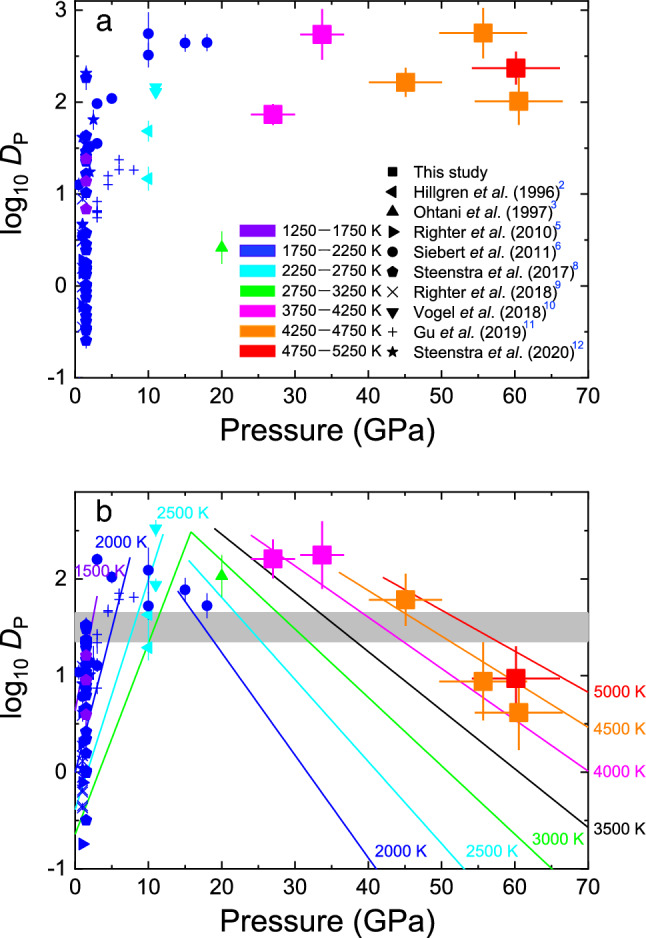
*D*_P_ (metal/silicate) (mole based) from this study and earlier experiments (only data with *f*O_2_ greater than ΔIW = -3, *nbo*/*t* < 3.7 and < 4 wt% C in metal, Supplementary Table S1). (**a**) Raw experimental data and (**b**) those adjusted to conditions for the Earth’s core formation (ΔIW = -2.3 and *nbo*/*t* = 2.57) using Eq. 2. The fitting of Eq. 2 was performed separately to data below 15 GPa and above, demonstrating changes in pressure and temperature effects on *D*_P_. The grey band in (**b**) indicates the geo-/cosmochemically estimated core/mantle distribution of phosphorus^23^.

The ${D}_{{\text{P}}}^{metal/silicate}$ values we obtained at 27–61 GPa are comparable to the majority of data previously reported at 10–20 GPa (Fig. 2a). These data cannot be fitted by a single equation (Eq. 2), since the positive pressure dependence that has been observed in a previous dataset to 8 GPa^11^ and to 18 GPa^6^ is not consistent with the present data obtained at higher pressures (Fig. 2b). We therefore performed fitting separately for data collected below and above 15 GPa. Indeed, when we employ a lower pressure for the fitting boundary, the *c* parameter (giving the pressure dependence in Eq. 2) for data in a higher pressure range remarkably changes from large negative to nearly zero because of the positive pressure dependence at low pressures (Supplementary Fig. S8). The fitting of Eq. 2 to earlier data below 15 GPa listed in Supplementary Table S1 yields *a* = − 3.12(171),* b* = 3835(2860), *c* = 594(112) and *d* = − 0.650(103); the large scatter of previous data reported below 1.5 GPa results in R^2^ = 0.55. On the other hand, the fitting to data for the higher pressure range above 15 GPa including the present ones provides *a* = 4.27(72),* b* = − 1464(883), *c* = − 212(49) and *d* = − 1.19(15) with R^2^ = 0.95, showing that the pressure dependence of *D*_P_ changes from positive to negative at 15 GPa (Fig. 2b). The *d* value, the *nbo*/*t* dependence, is found to be large negative even above 15 GPa, comparable to − 0.71(5) previously obtained from data collected up to 18 GPa^6^. Indeed, the definition of *nbo*/*t* assumes fourfold Si and is not applied to melts above ~ 10 GPa where Si is no longer tetrahedrally coordinated^20^. Nevertheless, the use of *nbo*/*t* is practical in this study since the effect of silicate melt composition is important at relatively low pressures^6^ and we realized that existing *D*_P_ data above 15 GPa from earlier studies^3,6^ and the present experiments are not well fitted without the effect of *nbo*/*t*. The present data give the mole-based *D*_P_ values ranging from 4 to 177 after adjusting to *f*O_2_ = IW-2.3 and *nbo*/*t* = 2.57 for a pyrolite composition, indicating that phosphorus is only modestly siderophile at typical conditions of Earth’s core metal segregation from silicate.

## Discussion

### Change in pressure dependence of ***D***_P_

Changes in the pressure evolution of metal–silicate partitioning have been suggested for a variety of elements. Cottrell et al*.*^16^ argued that the pressure dependence of the metal/silicate partition coefficient of tungsten alters from positive to negative around 3 GPa. Not only for W but also for Ni, Co, Mo, V and Cr, Rai and van Westrenen^17^ demonstrated that the pressure dependence found in data collected below 5 GPa is different from those observed in data obtained in a wider pressure range including > 5 GPa. For tungsten, recent XAFS measurements^21^ revealed an increase in the coordination number of W^6+^ from four to six, which can change the pressure dependence of its metal–silicate partitioning.

Since P^5+^ is a high-field strength element with relatively small ionic radius and high valence similar to W^6+^, the local structure of P^5+^ is close to that of W^6+^ in silicate melts and glasses. Ozawa et al.^21^ therefore supposed, based on the bond valence theory, that P^5+^ also increases its coordination number in a similar pressure range, leading to a remarkable volume reduction of PO_2.5_ in silicate melt. It can cause the volume of the left-hand side of Eq. 1 (PO_2.5_ + 2.5Fe) to be smaller than that of the right-hand side (P + 2.5FeO) (the pressure dependence of *D*_P_ therefore changes from positive to negative) above 15 GPa as we found in this study (Fig. 2b). Other high-field strength elements such as Mo and As may also undergo the coordination number increase in a comparable pressure range, which changes the pressure evolution of their metal–silicate partitioning. Furthermore, the observed changes in pressure dependence of the metal–silicate partitioning of W, Mo, V, and P and those of Ni and Co (Refs.^16,17^ and this study) might be explained by the onset and termination of the steep coordination number increase^21,22^, respectively.

### Core–mantle partitioning of phosphorus

**Figure 3:**
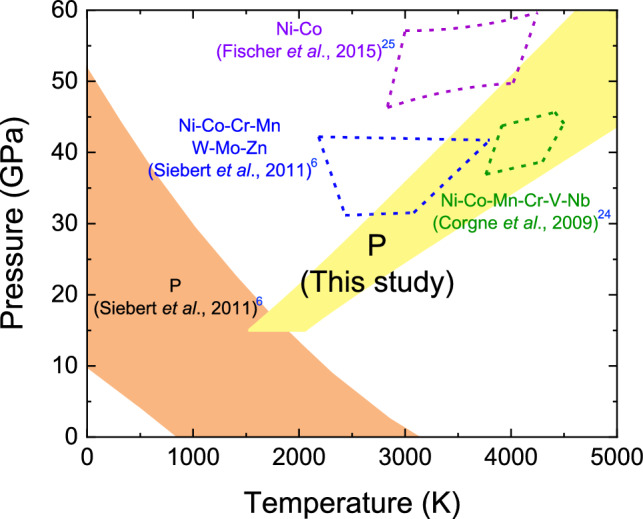
The range of *P*–*T* conditions at which metal/silicate partition coefficient that explains the Earth’s core/mantle distribution is obtained for each element. The yellow (this study) and orange areas^6^ for P; the blue area for Ni, Co, Cr, Mn, W, Mo and Zn (Ref.^6^); the green area for Ni, Co, Mn, Cr, V and Nb (Ref.^24^); the purple area for Ni and Co (Ref.^25^). The *P*–*T* range for P obtained in this study (yellow) overlaps with those for other siderophile elements, while the previous estimate based on low-pressure partitioning data (orange) does not.

Present experiments demonstrate that phosphorus is a modestly siderophile element under typical *P*–*T* conditions of the Earth’s core formation as a result of the change in pressure dependence of its metal–silicate partitioning around 15 GPa (Fig. 2b). Phosphorus concentrations have been estimated for the mantle (80–90 ppm) and for the bulk Earth (700–1200 ppm) with and without considering its volatility^13,14,18,19^. They give the P abundance in the core to be 2000–3700 ppm by weight from mass-balance calculations. These mantle and core concentrations show the apparent core/mantle distribution *D*_P_ (core/mantle) = 20 to 50 (mole based)^23^. Siebert et al.^6^ argued that the extrapolation of low-pressure data does not match such apparent *D*_P_ (core/mantle) under typical conditions of the Earth’s core formation, 30–60 GPa and 2500–4000 K (Fig. 3).

With the present new ${D}_{{\text{P}}}^{metal/silicate}$ at ΔIW = − 2.3 and *nbo*/*t* = 2.57 (Fig. 2b), we first consider conceptually simple, single-stage core formation scenarios that assume entire core–mantle chemical equilibration at a single *P*, *T* and *f*O_2_ condition. Figure 3 illustrates the range of *P–T* conditions under which the *D*_P_ (core/mantle) = 20–50 is obtained from the present high-pressure partitioning data (yellow area), along with that from earlier low-pressure data (orange area)^6^. The new data in this study overlaps with the range of 31–42 GPa and 2700–3800 K previously estimated on the basis of the core/mantle distributions of Ni, Co, Cr, Mn, W, Mo and Zn (Ref.^6^). It also overlaps with conditions for other pervious single-stage core formation models^24,25^.

**Figure 4:**
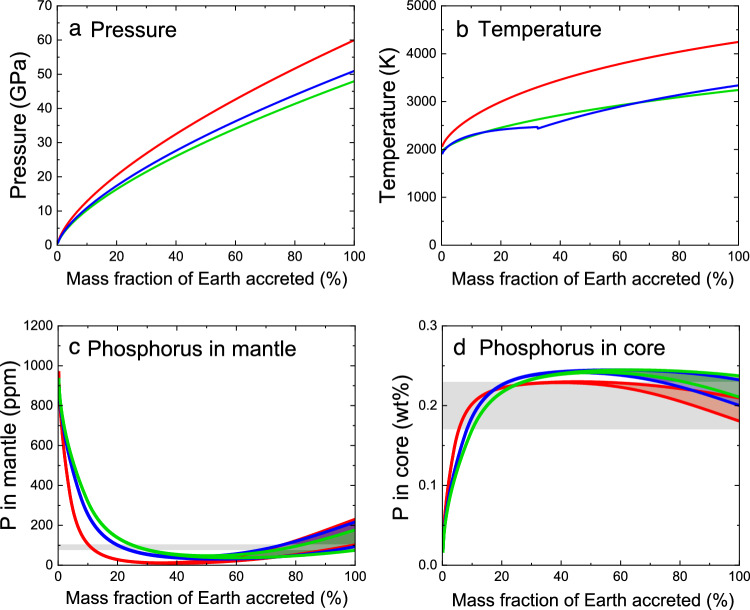
Evolutions of (**a**) pressure, (**b**) temperature, and phosphorus concentration in the (**c**) mantle and (**d**) core as a function of mass fraction of Earth accreted, based on the multi-stage core formation models S1 (red), R2 (green) and R3 (blue) reported by Tagawa et al.^26^ (Supplementary Table S2). Grey bands in (**c**) and (**d**) show geo-/cosmochemical estimates considering ± 15% uncertainty^19^. See Supplementary Fig. S9 for results using other models.

Next we calculate how much phosphorus is incorporated into the core when employing the multi-stage core formation models previously reported by Tagawa et al.^26^, which account for core mass, the mantle FeO, Ni and Co abundances, and ~ 700 ppm H_2_O in the bulk silicate Earth including oceans (see Supplementary Fig. 6 in Ref.^26^ for parameter space searched). In their models, metal–silicate partitioning took place by 1000 steps upon accretion of identical impactors, and the metal from each impactor equilibrated only with a limited fraction of silicate melt at the base of an existing magma ocean. We found that three out of nine models by Tagawa et al.^26^ explain P concentration observed in the mantle (Fig. 4, Supplementary Table S2). These three models show ~ 2000 ppm P in the core, consistent with the value calculated from the bulk Earth abundance that was estimated by considering the effect of its volatility^18,19^. Other six models result in the mantle P contents more than twice higher than observations as a consequence of relatively low ${D}_{{\text{P}}}^{metal/silicate}$ in the latter half of the Earth’s accretion, which derive from comparatively high final pressures at the bottom of a magma ocean, low temperatures or high *nbo*/*t* (Supplementary Fig. S9).

This study suggests that the core/mantle distribution of phosphorus, similar to those of other siderophile elements, is a natural consequence of core metal segregation in a deep magma ocean, where phosphorus is a modestly siderophile element. The Si effects proposed by Righter et al.^9^ may not have been serious for core–mantle partitioning in the Earth and the Mars.

## Methods

### High *P*–*T* experiments

Experiments were performed in a laser-heated diamond-anvil cell (DAC) with flat 300 μm culet anvils. Fe + 3wt%P foil (3N, Rare Metallic, 5–7 μm thick) was loaded into a ~ 130 µm hole at the centre of a pre-indented rhenium gasket, being sandwiched between the MORB glass powder. We used two MORB glass samples; both are similar in composition to that employed in previous experimental studies^26,27^ except titanium and calcium in SM1 (Table 2). Basaltic materials were employed because the solidus temperature is lower than that of pyrolite in the present experimental pressure range, which is helpful to secure a larger volume of silicate melt surrounding metallic liquid, and the chemical composition is closer to those of the standard silicates for the SIMS analyses. After loading the sample, the entire DAC was dried in a vacuum oven at 400 K for at least 24 h to eliminate moisture on the sample. Then, the sample was flushed with argon gas and subsequently compressed to a high pressure of interest in an argon atmosphere.

The sample was heated from both sides using a couple of 100 W single-mode Yb fibre lasers (IPG Photonics). The laser beam was converted to a flat-top distribution using beam shaping optics (New Focus). The laser spot size was around 40 μm. Heating duration was 10 s, which should be long enough to reach chemical equilibrium between liquid Fe and coexisting silicate melt when considering their sizes (~ 30 µm) (Fig. 1 and Supplementary Figs. S1 to S6). It has been discussed that such heating time scale (~ 10 s) is sufficient for chemical equilibrium in metal–silicate partitioning in a multi-anvil press in which the sample size is much larger^28,29^. Note that the diffusivity of phosphorus should be similar to those of Si and S in molten Fe (Ref.^30^) and is slightly smaller than that of silicon at ~ 1300 K and 1 bar but larger at > 3000 K in silicate melt^31^. Indeed, both liquid metal and molten silicate were found to be homogeneous in composition (Table 2), ensuring chemical equilibrium between them. Temperature was measured using a spectro-radiometric method. Experimental temperature was the one at the boundary between liquid metal and molten silicate. Sample pressure was determined from the Raman shift of the diamond anvil at ambient temperature after heating^32^. We considered the additional contribution of thermal pressure that has been estimated to be + 2.5 GPa per 1000 K. The overall errors in temperature and pressure may be ± 5% and ± 10%, respectively, according to Mori et al.^33^ in which such uncertainties were required for all of their experimental data to be consistent with each other.

### Chemical analyses with SIMS and EPMA

After recovering a sample from DAC at ambient condition, we prepared its cross-section at the centre of the laser-heated portion parallel to the compression/laser-heating axis by using a focused ion beam (FIB, FEI Versa 3D™). Textural and preliminary compositional characterizations were made on the sample cross section based on the X-ray elemental maps obtained by a field-emission-type scanning electron microscope (FE-SEM) and an energy-dispersive X-ray spectrometer (EDS) in the dual-beam FIB system (Fig. 1 and Supplementary Figs. S2 to S7). We then performed quantitative chemical analyses of quenched molten Fe and neighbouring silicate melt with FE-type electron probe microanalyzer (FE-EPMA, JXA-8530F, JEOL), except for phosphorus in silicate melt (Table 2). An acceleration voltage was 12 keV, a beam current was 15 nA and an electron beam diameter was 3 μm. We used Fe, Si, SiO_2_, Al_2_O_3_, MgO, CaSiO_3_, NaAlSi_3_O_8_, KAlSi_3_O_8_, KTiOPO_4_, Fe–0.84wt%C and Fe_3_C as standards, and LIF (Fe), LDE1 (O), PETJ (Si, Ca, P), TAP (Al, Na), TAPH (Mg), PETH (K) and LDE2H (C) as analysing crystals. The EPMA analytical totals for quenched silicate melt and liquid metal are low in some experiments because of the presence of small metal and oxide particles in silicate and metal, respectively, that were formed upon quenching temperature.

The phosphorus contents in silicate melts were determined with an isotope microscope system consisting of a stigmatic secondary ion mass spectrometry instrument (SIMS, CAMECA ims-1270e7) and a stacked CMOS-type active pixel sensor (SCAPS) at Hokkaido University^34^. This system provides projection images of secondary ions emitted from the sample surface. The images are converted to concentration maps by calibration curve methods^26,35^. ^133^Cs^+^ primary beam (15 keV, 30 nA) was irradiated over an approximately 100 μm × 100 μm area of the sample surface. A normal incidence electron gun was utilized for charge compensation of the analysis area. The contrast aperture was set 100 μm in diameter for projecting secondary ion imaging onto the SCAPS. Secondary ion images of ^31^P^−^ and ^28^Si^−^ were obtained by peak jumping of a sector magnet with accumulation times of 100 and 25 s, respectively (Fig. 1 and Supplementary Figs. S1 to S6). Differences in ^31^P^−^ intensities between metal and silicate melt are more than two orders of magnitude (Supplementary Figs. S1 to S6). The high intensity from the metals leaks into the surrounding silicate melts due to lens-flare or aberration effects of the ion optics of the isotope microscope. We carefully avoided the affected region to set regions of interest (ROIs) in the silicate melts which are free from the ^31^P^−^ intensity flare from the metals. The interference of ^30^Si^1^H^−^ on ^31^P^−^ was cut by the exit slit. Phosphorus concentrations in quenched silicate melts were quantified from the ^31^P^−^/^28^Si^−^ intensity ratio in ROIs (see areas surrounded by red lines in Fig. 1 and Supplementary Figs. S1 to S6), using a calibration curve established by standard glasses of Suprasil®, IND-G1, IND-G2 and FJ-G2^36^ (Supplementary Fig. S10) and the silicon content determined with an FE-EPMA. Errors in the phosphorus contents in silicate melts were calculated from the standard deviations of the ^31^P^−^/^28^Si^−^ intensity ratio in the ROI pixels (Table 1).

For runs #2 and #4, the sample cross sections were first analysed by an FE-EPMA. They were then further milled by an FIB until quenched liquid metal was lost, which provided a wide silicate melt area for subsequent SIMS analyses. The SIMS measurements were performed first on the sample cross sections from runs #1, 3, 5 and 6. The FE-EPMA analyses were made after re-polishing the sample surface with the FIB.

### Supplementary Information

Supplementary Information.

## Data Availability

The datasets obtained and analyzed during the current study are available from the corresponding author on reasonable request.
