# Supplementary material for: Pressure dependence of metal–silicate partitioning explains the mantle phosphorus abundance
Source: Sci Rep. 2024 Jan 12;14:1194. doi: 10.1038/s41598-024-51662-y (PMC10786851; doi:10.1038/s41598-024-51662-y)
Supplement: Supplementary file 1 — Supplementary Information. [file 41598_2024_51662_MOESM1_ESM.pdf]

Supplementary Information

Pressure dependence of metal-silicate partitioning explains the mantle phosphorus abundance

Nagi Ikuta[1]*, Naoya Sakamoto[2], Shoh Tagawa[3], Kei Hirose[1,3], Yutaro Tsutsumi[1], Shunpei Yokoo[1] & Hisayoshi Yurimoto[2,4]

[1]Department of Earth and Planetary Science, The University of Tokyo, Tokyo 113-0033, Japan.
[2]Creative Research Institution (CRIS), Hokkaido University, Sapporo, Hokkaido 001-0021, Japan.
[3]Earth-Life Science Institute, Tokyo Institute of Technology, Tokyo 150-8550, Japan
[4]Department of Natural History Sciences, Hokkaido University, Sapporo, Hokkaido 060-0810, Japan.

Supplementary Text

Activities of elements in carbon-bearing metal. The carbon content in quenched liquid metal ranges from 0.82 to 2.3 wt% (Table 2). The presence of carbon diminishes the molar fractions of iron and phosphorus in metal, which apparently increases the oxygen fugacity $\Delta\mathrm{IW}{\sim}2\ \log_{10}\left(x_{\mathrm{FeO}}^{silicate}/x_{\mathrm{Fe}}^{metal}\right)$ and changes the exchange coefficient $K_{\mathrm{D}}^{\mathrm{O}} = x_{\mathrm{Fe}}^{metal}x_{\mathrm{O}}^{metal}/x_{\mathrm{FeO}}^{silicate}$ for the reaction $\mathrm{FeO}^{silicate} = \mathrm{Fe}^{metal} + \mathrm{O}^{metal}$. Tagawa *et al.* (2021)[26] calculated the $K_{\mathrm{D}}^{\mathrm{O}}$ values using molar fractions of iron and oxygen in metal, with and without considering the presence of hydrogen and carbon, and found that the latter results are consistent with those obtained in the (H, C)-free system[25]. It suggests that both hydrogen and carbon do not have colligative properties in iron solvent likely because small H and C atoms are incorporated into liquid Fe interstitially rather than substitutionally unlike other, larger atoms[15]. Therefore, in this study, we approximate the activity of element *i* in metal as;

$$x_i' = \frac{N_i}{\sum_{k\neq C} N_k}\ . \qquad \text{(S1)}$$

The same procedure was applied when we obtain mole-based *D* values from earlier experimental data (Fig. 2).

Supplementary Tables

	P (GPa)	*T* (K)	ΔIW	*nbo*/*t*	D_P (weight based)	D_P (mole based)
Hillgren *et al.* (1996)[2]	10	2273	-1.33	0.62	48.0	48.4
	10	2273	-2.32	2.80	12.9	14.7
Ohtani *et al.* (1997)[3]	20	2773	-1.80	3.41	2.2	2.6
Righter *et al.* (2010)[5]	1	1773	-1.87	2.24	0.1	0.1
	1	1873	-2.15	2.47	0.5	0.6
	1	1973	-2.15	2.52	1.3	1.4
	1	2073	-2.18	2.88	1.7	1.9
Siebert *et al.* (2011)[6]	3	2123	-2.28	2.88	85.5	96.1
	3	2123	-2.53	2.33	29.2	35.4
	2	2123	-2.63	2.64	26.6	32.3
	1	2123	-2.16	2.69	2.9	3.5
	0.5	2123	-2.28	2.44	10.5	12.6
	10	2173	-2.70	2.12	271.4	325.0
	10	2123	-2.69	2.32	460.6	555.3
	5	2123	-2.61	3.13	89.0	109.4
	15	2173	-2.41	2.05	368.9	439.1
	18	2173	-2.49	1.99	370.3	444.0
Steenstra *et al.* (2017)[8]	1.5	1783	-1.16	2.16	1.5	1.5
	1.5	1783	-1.45	2.03	3.7	3.8
	1.5	1783	-1.31	1.98	1.6	1.6
	1.5	1783	-1.17	2.01	0.6	0.6
	1.5	1783	-0.96	2.63	0.4	0.4
	1.5	1783	-1.09	2.22	0.1	0.1
	1.5	1783	-1.51	1.83	2.4	2.5
	1.5	1783	-1.26	2.31	1.7	1.7
	1.5	1783	-1.28	2.02	0.9	0.9
	1.5	1783	-1.10	2.31	1.6	1.6
	1.5	1883	-1.31	2.59	1.8	1.8
	1.5	1883	-1.20	2.61	1.4	1.3
	1.5	1683	-1.97	0.73	23.6	24.1
	1.5	1683	-1.41	1.03	6.8	6.8
	1.5	1683	-1.43	1.01	14.0	13.8
	1.5	1883	-1.82	1.59	22.1	22.6
	1.5	1883	-2.61	1.22	176.6	184.1
	1.5	1883	-1.72	1.60	10.5	10.3
	1.5	1883	-2.04	1.65	16.1	16.6
	1.5	1883	-2.21	1.41	*41.6*	43.0
	1.5	1883	-2.04	1.70	24.9	25.8
	1.5	1883	-1.95	1.72	28.5	29.0
	1.5	1883	-1.65	3.09	0.3	0.3
	1.5	1883	-1.81	3.18	0.2	0.3
	1.5	1883	-2.26	2.50	0.9	1.0
	1.5	1883	-2.21	2.90	0.9	1.0
	1.5	1883	-2.56	2.68	2.6	2.8
	1.5	1883	-2.47	2.64	0.4	0.5
	1.5	1883	-1.21	3.46	0.3	0.3

	1.5	1883	-1.56	3.44	0.8	0.7
	1.5	1883	-1.44	3.21	1.0	1.0
Righter *et al.* (2018)[9]	1	1873	-2.25	3.31	3.5	3.6
	1	1873	-2.53	3.16	2.6	3.1
	1	1873	-2.10	2.37	0.9	1.0
	1	1873	-2.24	2.57	1.4	1.6
	1	1873	-2.53	2.53	2.8	3.1
	1	1873	-2.77	1.94	8.0	8.8
	1	1873	-2.08	2.52	0.3	0.4
	1	1873	-2.29	2.30	0.6	0.6
	1	1873	-2.47	2.34	0.9	1.0
	1	1873	-2.84	2.35	3.7	4.3
Vogel *et al.* (2018)[10]	11	2614	-2.81	3.21	125.8	145.4
	11	2605	-2.41	3.41	113.9	128.6
Gu *et al.* (2019)[11]	3	1973	-2.43	3.63	5.6	6.6
	3	1973	-2.11	2.67	5.6	6.3
	3	2073	-1.74	2.27	7.9	8.3
	3	1973	-2.49	3.03	5.6	6.4
	3	1973	-2.13	2.84	5.0	5.5
	4.5	1973	-1.99	2.68	14.0	15.8
	4.5	2073	-1.77	2.43	12.1	12.7
	6	1973	-1.94	2.52	21.7	23.6
	6	2073	-1.80	2.52	17.7	18.3
	8	2173	-1.74	2.34	17.3	18.2
Steenstra *et al.* (2020)[12]	1	1883	-2.12	1.14	35.2	41.4
	1.5	1883	-2.01	1.29	33.2	35.5
	2	1883	-1.86	1.36	16.3	17.4
	2.5	1883	-2.15	1.24	59.7	64.3
	1.5	1883	-2.97	0.94	214.1	205.8
	1	1883	-2.11	1.24	4.3	4.7

Supplementary Table S1. Previous experimental data on metal-silicate partitioning of phosphorus.

**Model**	**Core mass fraction**	**Earth silicate**					**Earth core**					**Difference from target values**[a]
		**FeO**	**Ni**	**Co**	**H_2O**	**P**	**O**	**Si**	**S**	**H**	**P**	
		**wt%**	**ppm**	**ppm**	**ppm**	**ppm**	**wt%**	**wt%**	**wt%**	**wt%**	**wt%**	
S1	0.307	8.25	2101	108	720	162	2.75	6.38	2.05	0.56	0.20	0.0097
S2	0.282	8.35	2131	115	735	266	1.59	1.84	2.24	0.33	0.19	0.0357
S3	0.285	8.14	2302	119	715	287	1.63	2.29	2.21	0.33	0.18	0.0626
F1	0.329	8.75	1730	93	723	318	1.92	11.37	1.92	0.35	0.15	0.0356
F2	0.301	7.95	2160	100	747	319	0.91	5.79	2.09	0.33	0.16	0.0179
F3	0.293	8.24	2043	99	729	283	0.86	4.46	2.15	0.32	0.18	0.0151
R1	0.341	8.42	1976	170	687	341	1.85	13.16	1.85	0.27	0.14	0.3912
R2	0.279	8.26	1834	137	736	120	0.59	1.35	2.26	0.28	0.23	0.1182
R3	0.279	8.20	2188	152	745	149	0.61	1.39	2.26	0.29	0.22	0.2334
Present Earth	0.325	8.05	1960	105	>690	90	-	-	-	-	0.2	-

Supplementary Table S2. The distributions of phosphorus based on the earlier multi-stage core formation models by Tagawa *et al.* (2021)[26]. [a]Defined as $\Sigma((\text{calculated value})/(\text{present Earth value})-1)^2$ for core mass fraction and mantle concentrations of FeO, Ni and Co.

Supplementary Figures

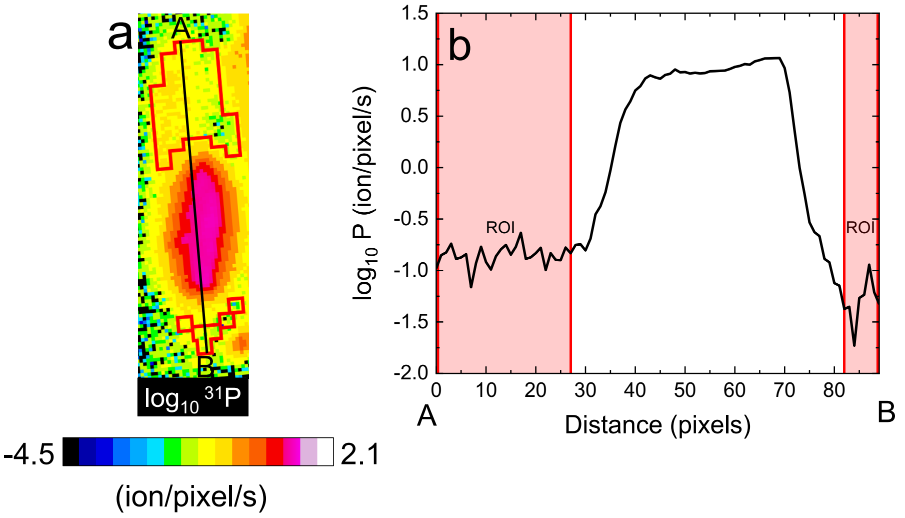

Supplementary Figure S1. Secondary $\log_{10}$ $^{31}P^{-}$ ion image **(a)** and its line profile **(b)** (from A to B in **(a)**) obtained in run #3. See Fig. 1 for other images. Portions surrounded by red lines (ROI) in the silicate melt **(a)** is free from the lens-flare effects to measure the P content. Variations in P concentration in the ROI correspond to 24–170 ppm, and the concentration in the silicate melt is determined to be 87±36 ppm.

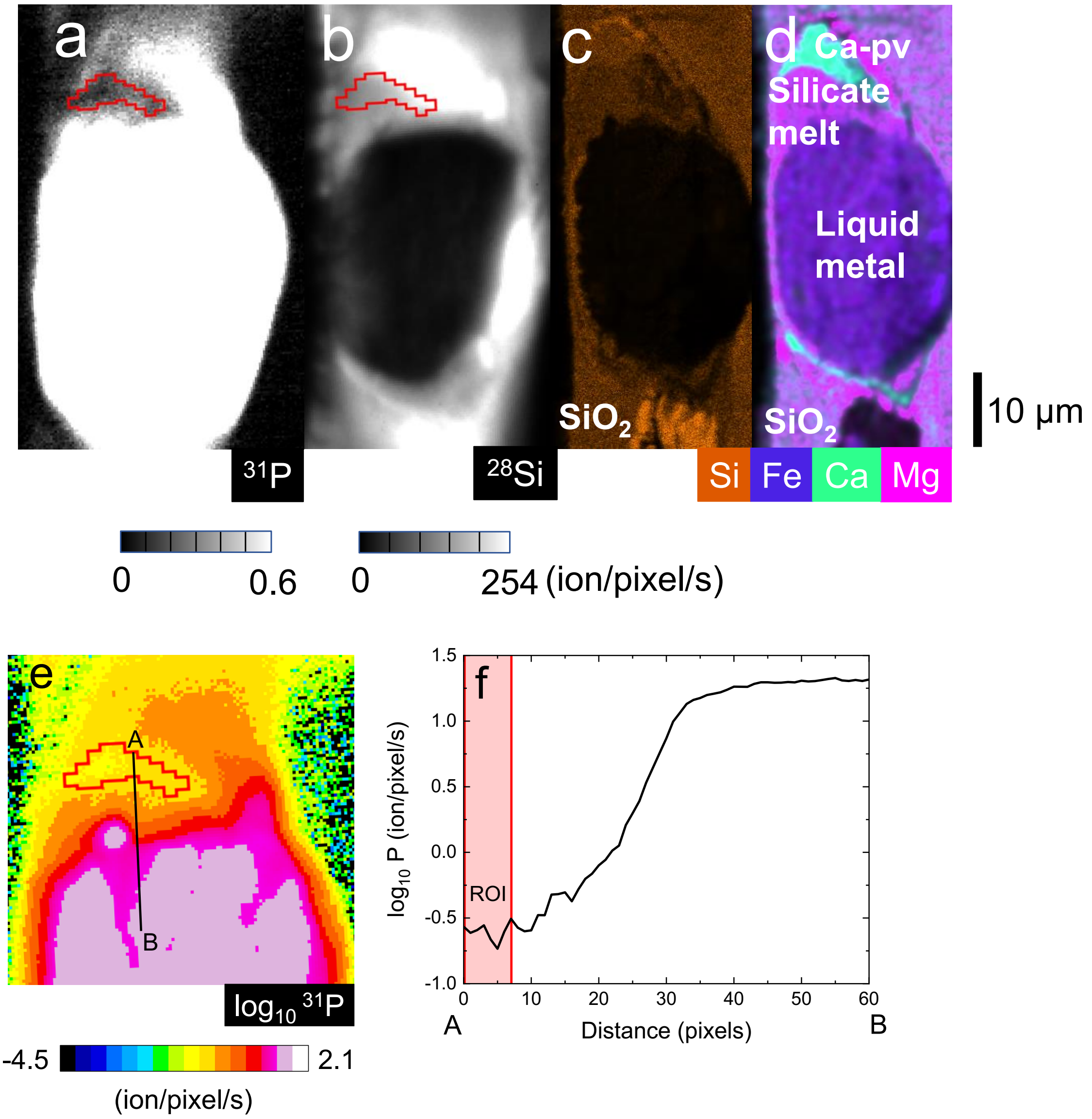

Supplementary Figure S2. Secondary ion images for **(a)** $^{31}P^-$ and **(b)** $^{28}Si^-$, and EDS X-ray maps for **(c)** Si and **(d)** Fe/Ca/Mg of a sample cross section obtained in run #1. Quenched liquid metal was surrounded by silicate melt. $CaSiO_3$-rich perovskite (Ca-pv) and the SiO_2 phase were present outside the silicate melt. The liquid metal area of **(a)** is apparently larger than that of **(b)** because of lens-flare effects of the secondary ion optics due to extremely high ^{31}P intensities from liquid metal (maximum 49 ion/pixel/s). The effects are relatively small upwards. The $\log_{10}$ $^{31}P^-$ image **(e)** and its line profile **(f)** (from A to B in **(e)**) show that a portion surrounded by red line (ROI) in the silicate melt **(a, b)** is free from the lens-flare effects to measure the P content. Variations in P concentration in the ROI correspond to 89–191 ppm, and the concentration in the silicate melt is determined to be 133±45 ppm.

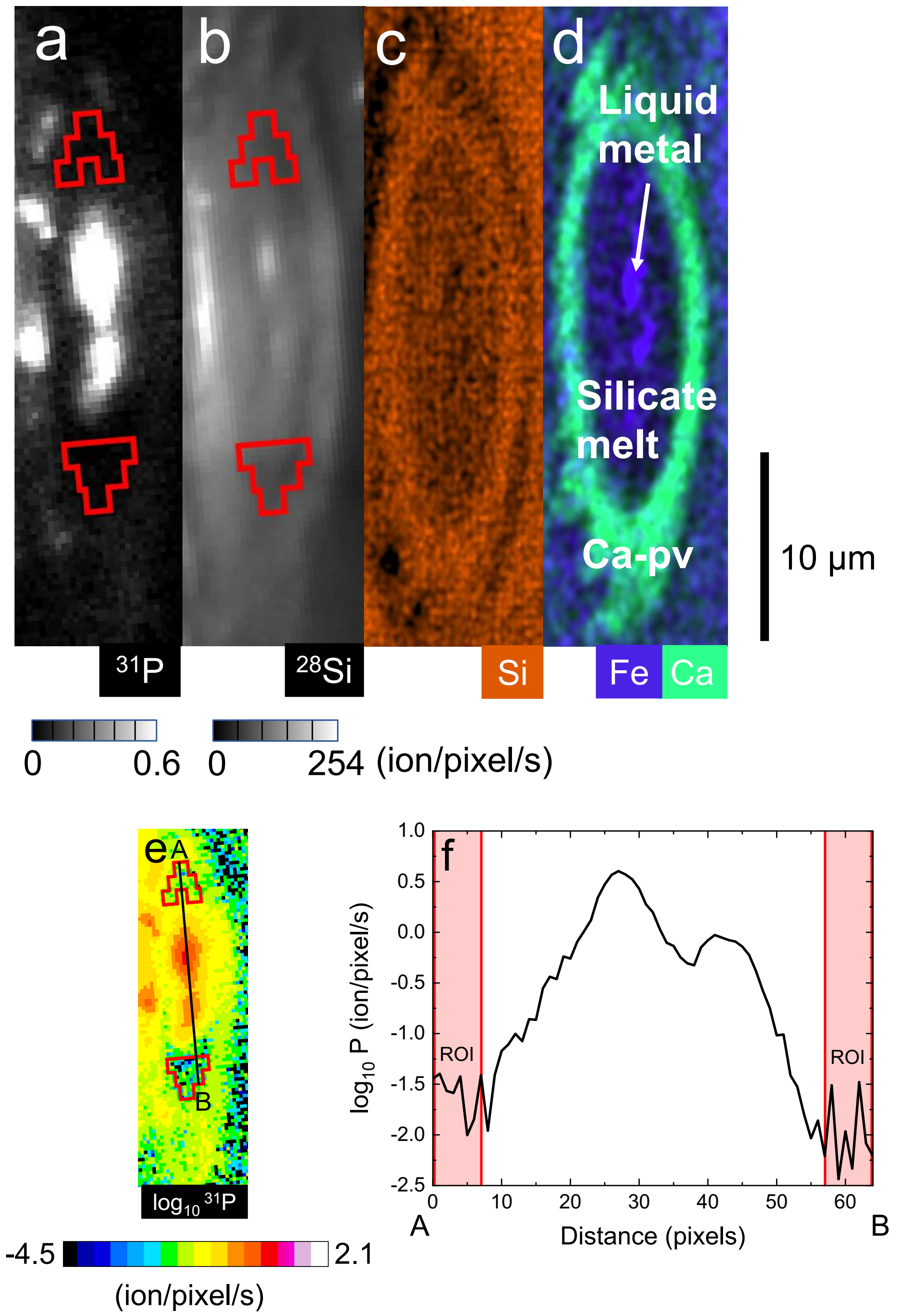

Supplementary Figure S3. Secondary ion images for **(a)** $^{31}P^{-}$ and **(b)** $^{28}Si^{-}$, and EDS X-ray maps for **(c)** Si and **(d)** Fe/Ca of a sample cross section obtained in run #2. Quenched molten metal was removed after EPMA analyses by polishing with an FIB to obtain a wider area of the silicate melt for SIMS measurements, while some was left at the centre (see Fe enrichment in **(d)**). The strong P signals **(a)** are from the liquid metal. The areas surrounded by red lines (ROI) in **(a, b)** were chosen to avoid the $^{31}P^{-}$ signals from the liquid metal to obtain P concentration in the silicate melt (see the $\log_{10}$ $^{31}P^{-}$ image **(e)** and its line profile **(f)**). Variations in P concentration in the ROI correspond to 14–77 ppm, and the concentration in the silicate melt is determined to be 39±17 ppm.

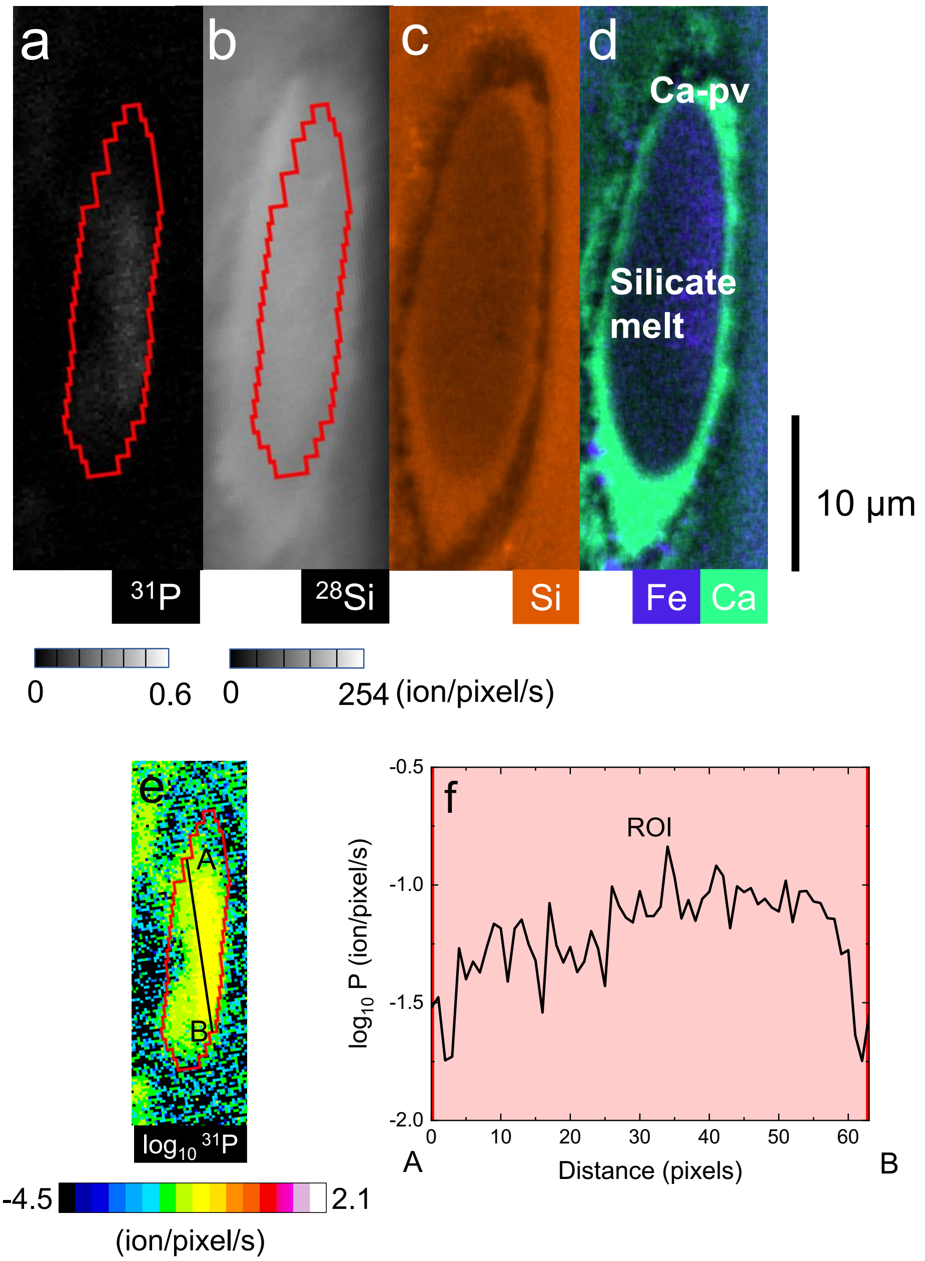

Supplementary Figure S4. Secondary ion images for **(a)** $^{31}P^-$ and **(b)** $^{28}Si^-$, and EDS X-ray maps for **(c)** Si and **(d)** Fe/Ca of a sample cross section obtained in run #4. Quenched molten metal was removed after EPMA analyses by polishing with an FIB such that a wider area of the silicate melt was measured by SIMS. The silicate melt region is surrounded by Ca-pv **(c, d)**, and its P concentration was determined from the ROI shown by the red lines in **(a**, **b)**. The $\log_{10}$ $^{31}P^-$ image **(e)** and its line profile **(f)** are also given. Variations in P concentration within the ROI that appear in **(a)** corresponds to 2–97 ppm, and the concentration in the silicate melt is determined to be 42±27 ppm.

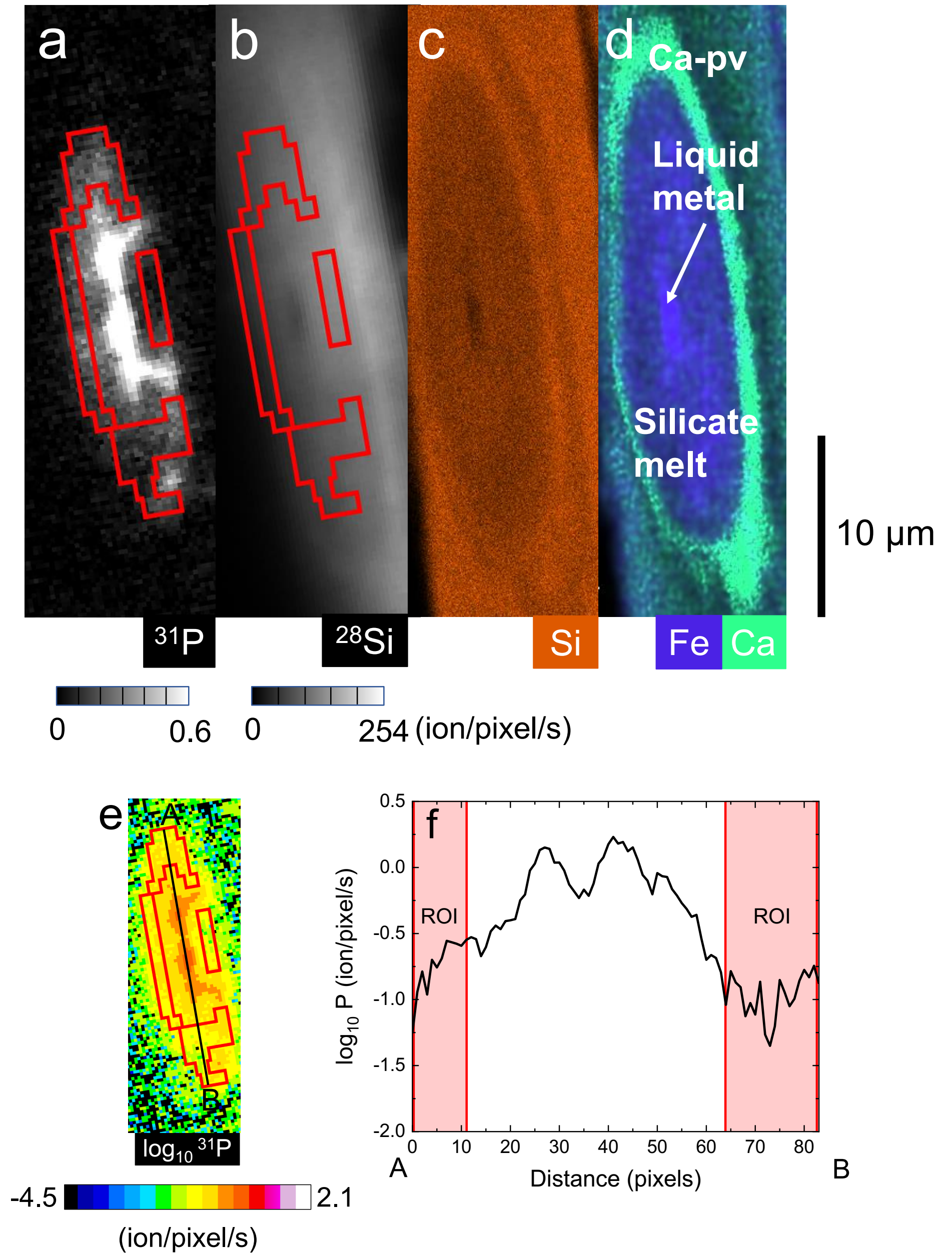

Supplementary Figure S5. Secondary ion images for **(a)** $^{31}P^-$ and **(b)** $^{28}Si^-$ and EDS X-ray maps for **(c)** Si and **(d)** Fe/Ca of a sample cross section obtained in run #5. A small amount of metal was present at the centre portion of the silicate melt **(c, d)**, showing the enrichment in P **(a)**. The areas surrounded by red lines (ROI) in **(a, b)** were chosen to avoid $^{31}P^-$ signals from the liquid metal to obtain P concentration in the silicate melt (see the $\log_{10}$ $^{31}P^-$ image **(e)** and its line profile **(f)**). Variations in P concentration in the ROI correspond to 41–764 ppm, and the concentration in the silicate melt is determined to be 206±106 ppm. EPMA analyses of quenched molten metal were made on a different cross section obtained by further polishing with an FIB.

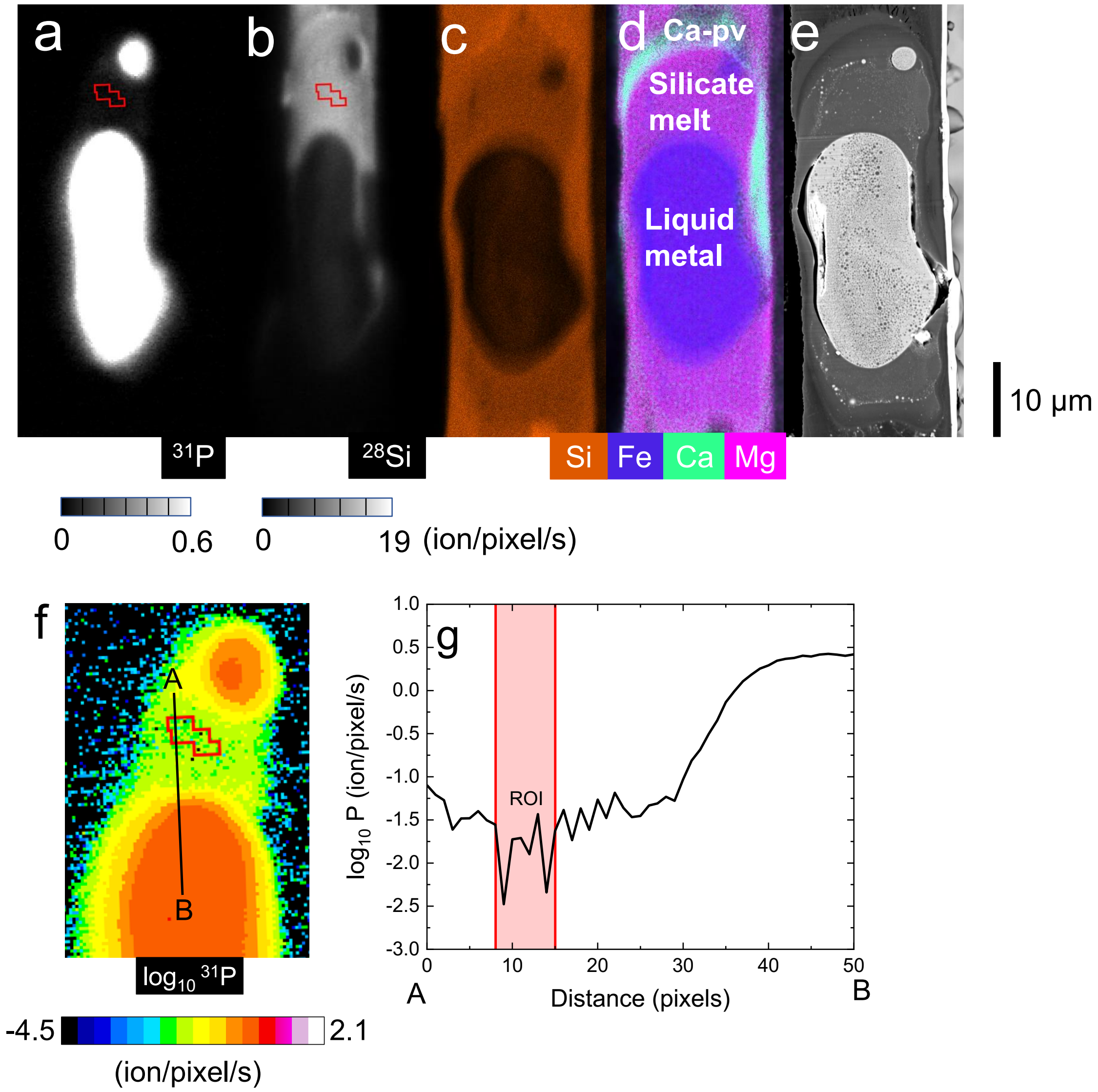

Supplementary Figure S6. Secondary ion images for **(a)** $^{31}P^-$ and **(b)** $^{28}Si^-$, EDS X-ray maps for **(c)** Si and **(d)** Fe/Ca/Mg, and **(e)** back-scattered electron image of a sample cross section obtained in run #6. Quenched liquid metal was surrounded by silicate melt, and Ca-pv was present outside the silicate melt. The liquid metal area in **(a)** is apparently larger than that of **(b)** because of the lens-flare effects of the secondary ion optics due to extremely high ^{31}P intensities from liquid metal (maximum 7.3 ion/pixel/s). The effects are relatively small upwards. The $\log_{10}$ $^{31}P^-$ image **(f)** and its line profile **(g)** (from A to B in **(f)**) show that a portion surrounded by red line (ROI) in the silicate melt **(a, b)** is free from the lens-flare effects to measure the P content. Variations in P concentration in the ROI correspond to 207–303 ppm, and the concentration in the silicate melt is determined to be 266±38 ppm.

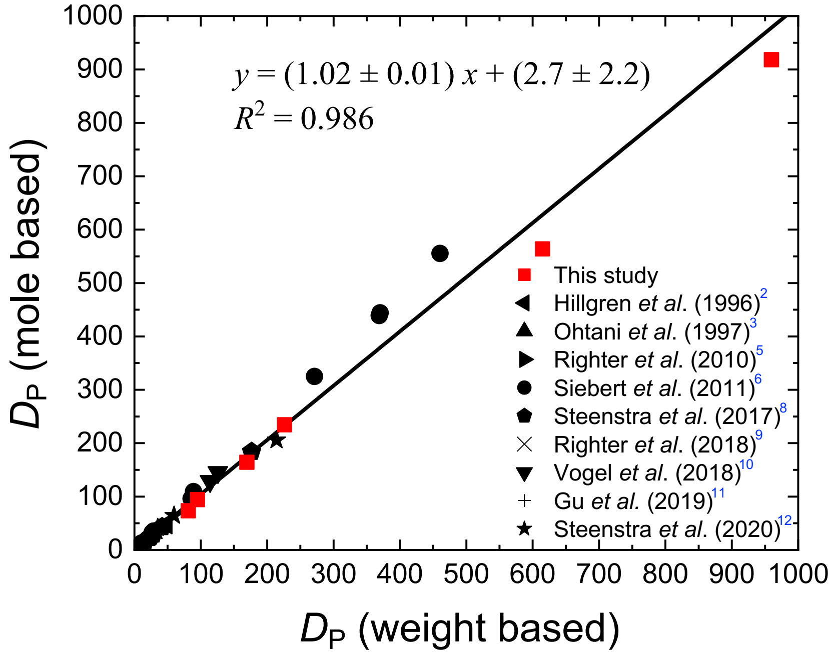

Supplementary Figure S7. Comparison between weight-based and mole-based metal/silicate partition coefficients, D_P reported in this study and earlier experiments.

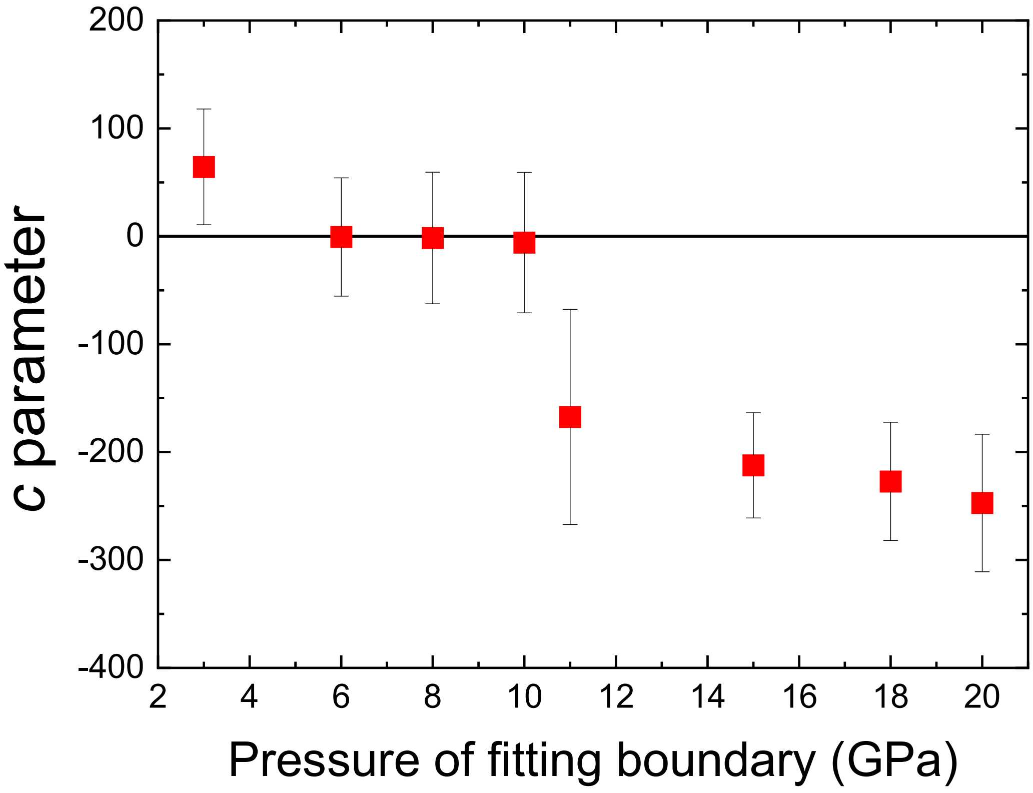

Supplementary Figure S8. Change in the *c* parameter obtained by fitting Eq. 2 to D_P data in a higher pressure range when varying the lower bound of the pressure range for fitting. Note that the *c* parameter, indicating the pressure dependence of D_P, becomes large negative and has the smallest error when fitting to the data collected above 15 GPa, while it is close to zero when including data at lower pressures because of a positive pressure dependence below 11 GPa (see Fig. 2b).

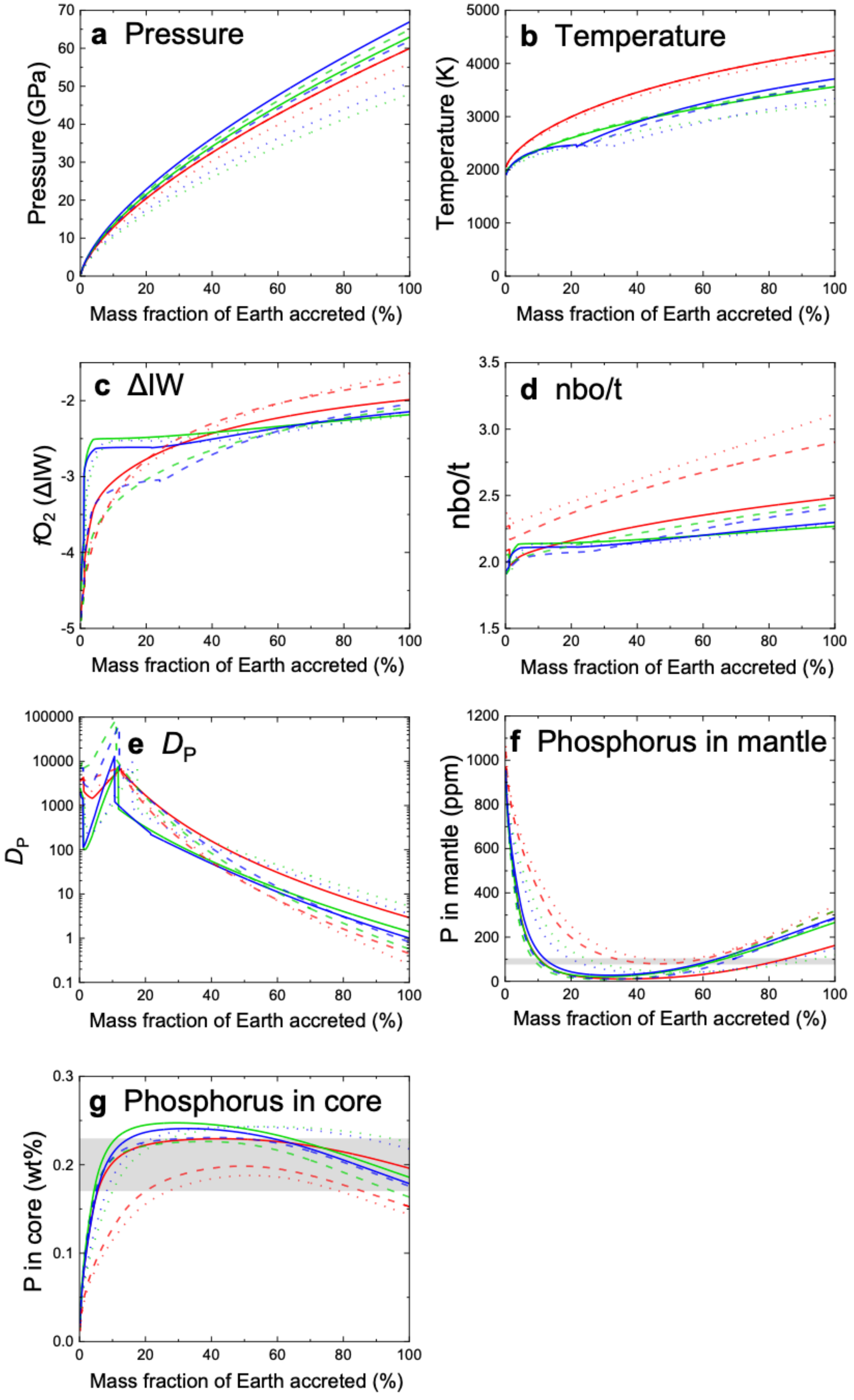
a Pressure
Pressure (GPa)
Mass fraction of Earth accreted (%)
b Temperature
Temperature (K)
Mass fraction of Earth accreted (%)
c ΔIW
fO2 (ΔIW)
Mass fraction of Earth accreted (%)
d nbo/t
nbo/t
Mass fraction of Earth accreted (%)
e DP
DP
Mass fraction of Earth accreted (%)
f Phosphorus in mantle
P in mantle (ppm)
Mass fraction of Earth accreted (%)
g Phosphorus in core
P in core (wt%)
Mass fraction of Earth accreted (%)

Supplementary Figure S9. Evolutions as a function of mass fraction of Earth accreted in multi-stage core formation models proposed by Tagawa *et al.* (2021)[26]. (**a**) Pressure, (**b**) temperature, (**c**) fO_2, (**d**) *nbo*/*t* of silicate, and (**e**) metal/silicate partition coefficient (mole based) for chemical reaction at each step, and phosphorus concentrations in the mantle (**f**) and the core (**g**). They were calculated for nine different combinations of partitioning data (solid curves, S1–S3; broken curves, F1–F3; dotted curves, R1–R3) and *P*-*T* path (red, T_{MOB1}; green, T_{MOB2}; blue, T_{MOB3}). Grey bands in (**f**) and (**g**) show the present Earth values considering ±15% uncertainty[19]. See Supplementary Table 3 in Tagawa *et al.* (2021)[26] for each parameter set.

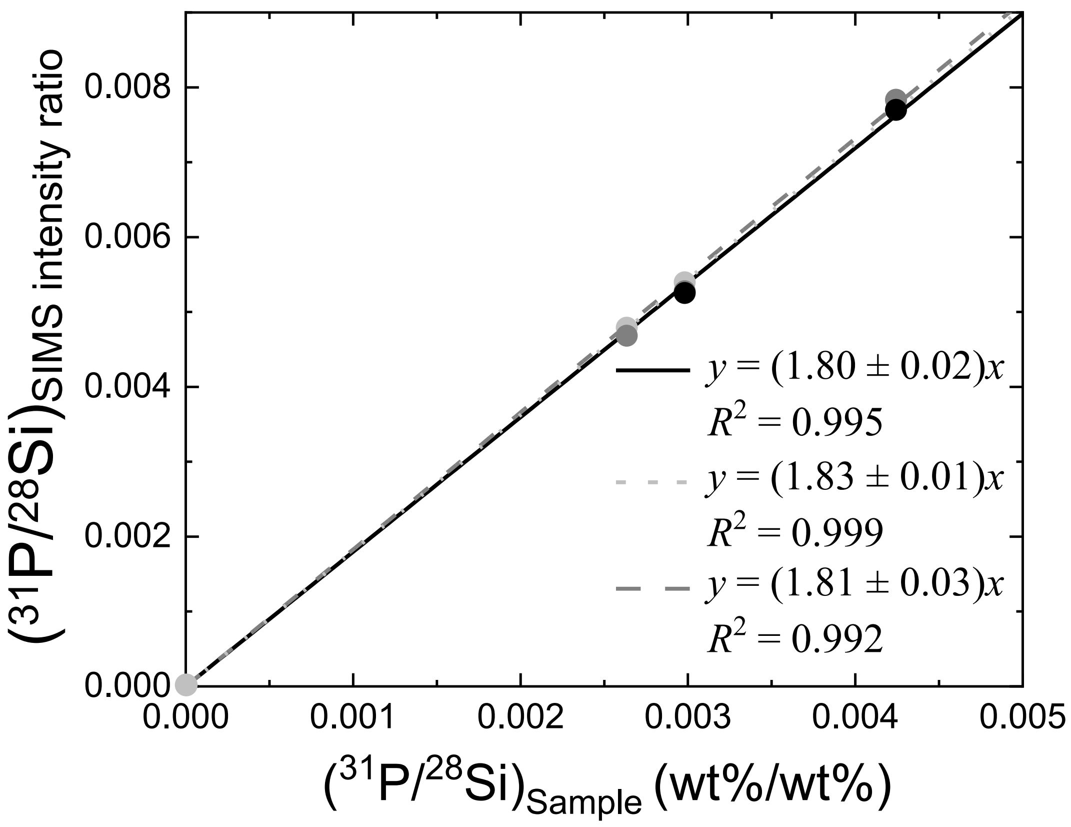

Supplementary Figure S10. Calibration curves for $^{31}P/^{28}Si$ based on the SIMS analyses of standard glasses[36]. Black symbols and solid line are for run #1, light-grey symbols and dotted line are for runs #2–5, and dark-grey symbols and broken line are for run #6.
